# The Long-Term Impact of Rotavirus Vaccines in Korea, 2008–2020; Emergence of G8P[8] Strain

**DOI:** 10.3390/vaccines9040406

**Published:** 2021-04-20

**Authors:** Kwang gon Kim, Hye-young Kee, Hye jung Park, Jae Keun Chung, Tae sun Kim, Min Ji Kim

**Affiliations:** Health and Environment Research Institute of Gwangju, Gwangju 61954, Korea; kkg1229@korea.kr (K.g.K.); vetkhy@korea.kr (H.-y.K.); sdgenie@korea.kr (H.j.P.); jkchung@korea.kr (J.K.C.); kts2877@korea.kr (T.s.K.)

**Keywords:** rotavirus, genotype, Rotarix, RotaTeq, G8P[8]

## Abstract

This study evaluated the long-term impact of rotavirus vaccination on prevalence, seasonality, and genotype distribution in Gwangju, Korea for 13 seasons. Rotavirus was identified using ELISA and then sequenced for G and P genotypes by Reverse Transcription Polymerase Chain Reactions for diarrhoeagenic patient specimens from local hospitals between January 2008 and August2020. Of 26,902 fecal samples, 2919 samples (10.9%) were ELISA positive. The prevalence declined from 16.3% in pre-vaccine era to 5.4% in post-vaccine era. In the pre-vaccine period, G1P[8] was the most common genotype, followed by G2P[4], G3P[8], and G9P[8], etc. In the transitional period, the proportion of G2P[4] became the dominant genotype and G1P[8] was still commonly identified. In contrast, the novel genotype G8P[8] was predominant in the post-vaccine period. Meanwhile, G2P[4] and G8P[8] were major genotypes in both Rotarix and RotaTeq groups. The substantial decline of G1P[8] prevalence, reemergence of G1P[8], G3P[8], and G2P[4] rotavirus strains, and surge of the rare G8P[8] after vaccine introduction were interesting points to note. The continuous surveillance on the genotypes of RV will be needed to understand rotavirus epidemiology and their evolutionary patterns, as caution is required when interpreting temporal changes in RV genotype dynamic.

## 1. Introduction

Rotavirus (RV) is one of the leading causes of gastroenteritis in young children and many other animal species [1]. Although there is a large discrepancy in incidence and mortality between high- and low-income countries, RV infection is responsible for nearly 130,000 deaths annually with a considerable economic burden [2]. The genome of RV consists of 11 double-stranded RNA (dsRNA) surrounded by a three-layered icosahedral protein capsid. The RNA segments encode six structural viral proteins (VP1- VP4, VP6, and VP7) and six non-structural viral proteins (NSP1-6) [3].

Ten RV species (Group A–J) have been identified on the basis of sequence and antigenic differences of VP6 encoding segment [4]. Group A Rotavirus (RVA) is further classified into different genotypes based on VP7 and VP4 encoding segments. To date, 32 G genotypes and 47 P genotypes of group A Rotavirus were identified based on VP7 (G, glycoprotein) and VP4 (P, protease-cleaved protein) segments, although only a few combinations of G and P types such as G1P[8], G2P[4], G3P[8], G4P[8], G9P[8], and G12P[8] predominate worldwide [4]. Previous studies revealed that G1P[8] viruses consistently represent the most common RV serotypes globally prior to the introduction of RV vaccines [3,5].

Two RV vaccines Rotarix (monovalent, live-attenuated G1P[8] strains of human RV, licensed in September 2007) and RotaTeq (pentavalent, human-bovine re-assortantG1-G4, and P[8] strain, licensed in July 2008) were licensed for use in Korea [6]. Compared to the other high-income countries that achieved high vaccine coverage soon after the introduction, the coverage has slowly increased in Korea, as RV vaccines have not been implemented into National Immunization Program (NIP). Consequently, RV vaccine uptake rate grew slowly; 5.5%, 26.1%, 34.2%, and 45.2% in 2011, 2012, 2013, and 2014, respectively [7]. The vaccination coverage reached a plateau of up to 80–85% since 2015 [6]. The impact of RV vaccine in many countries such as Finland, Belgium, Spain, Austria, and Brazil, where RV vaccine had been implemented nationally, were evident both in epidemiologic changes of RV, strain evolution through selective pressure, and economic burden, etc.[5,8,9,10,11].

Whether the changes in RV genotypes represent natural/seasonal shifts or selection pressure related RV vaccine is still unclear, and the emergence of novel genotypes have been observed in recent years, suggesting the necessity of continuing surveillance of RV prevalence and genotypes [4,12]. Therefore, the purpose of this study was to evaluate the long-term impact of rotavirus vaccination on prevalence, seasonality, and genotype distribution in Gwangju, Korea for 13 seasons. We analyzed laboratory data from January 2008 to August 2011 (pre-vaccine), September 2011 to August 2015 (transitional), and from September 2015 to August 2020 (post-vaccine).

## 2. Materials and Methods

After diagnosis of gastroenteritis by pediatrician, stool specimens of patients, whose consent sheet had been secured for laboratory testing, were collected, anonymized, then submitted for routine bacterial and viral diagnostic testing to Health and Environment Research Institute (HERI) from 12 local hospitals from 2008 to 2020 in Gwangju, Korea. The data covers the southeast region of Korea, as major tertiary care centers as well as primary hospitals are located at Gwangju.

Rotavirus was initially identified using enzyme-linked immunosorbent assay (ELISA) with VP6 specific antibody. Approximately 10% suspensions of stool samples in phosphate-buffered saline (PBS) were prepared and 100 μL of supernatants were used for ELISA after centrifugation at 3000rpm for 15min. BioTracer Rotavirus ELISA kit (Biofocus, Uiwang, Korea) and RIDASCREEN Rotavirus (R-Biopharm, Darmstadt, Germany) were used. Then, viral RNA was extracted from RV positive samples using a QIAamp Viral RNA mini kit (Qiagen, Hilden, Germany) and QIAsymphony DSP Virus/Pathogen Mini Kit (Qiagen, Hilden, Germany) according to the manufacturer’s instructions. Reverse transcription polymerase chain reactions (RT-PCRs) were performed after RNA denaturation step for 5minat 95 °C to detect RV VP7 and VP4 sequences. Forward primer (con3, 5′ -TGG CTT CGC TCA TTT ATA GAC A-3′) and reverse primer (con2, 5′ -ATT TCG GAC CAT TTA TAA CC-3′) were used for VP4 gene, forward primer (VP7-F, 5′ -ATG TAT GGT ATT GAA TAT ACC AC-3′) and reverse primer (VP7-R, 5′ -AAC TTG CCA CCATTT TTTCC-3′) were used for VP7 gene, which is expected to be 876bp, 881bp, respectively. Positive RT-PCR products were further analyzed by sequencing reaction with the same primers as in RT-PCR.

The available data about samples were age, gender, sampling date, vaccination status, etc. Vaccination status was included since 2013. Age groups (<5 years, 5–9 years, 10–16 years, and >17 years as adults) were used to show rotavirus positivity by season. About 25% RV positive samples from 2008, 2008–11, and 2012–13 RV seasons, sorted with respect to age, gender, and date of sample collection, were included for VP4 and VP7 genotyping, and also non-typable RV cases were excluded from the final analysis. We defined the pre-vaccine (from January 2008 to August 2011), transitional (from September 2011 to August 2015), and post-vaccine (from September 2015 to August 2020) periods, as the vaccine uptake increased progressively from 5.5% to 80% in the transitional period. Statistical analysis was conducted using SPSS 20.0 (IBM corp, Armonk, NY, USA). χ^2^ analysis was used to compare differences in genotype by vaccination period and prevalence by period, etc. *p*-values ≤ 0.05 were considered as statistically significant.

## 3. Results

**Figure 1:**
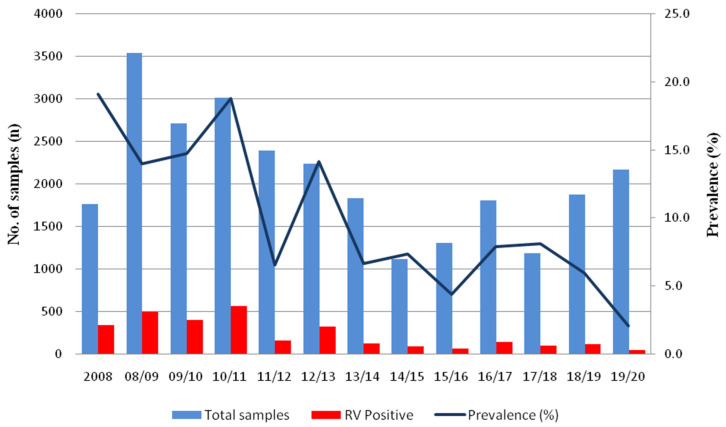
Absolute frequencies of patient samples, rotavirus-positive samples investigated, and prevalence of rotavirus throughout the study period in Korea, 2008–2020.

During thirteen RV seasons from 2008 to 2020, a total of 26,902 fecal samples of patients with GI symptoms were received from collaborating hospitals in Gwangju, Korea. Of these, 2919 samples (10.9%) were ELISA positive for RV. The mean age of RV positive patients was 3.8 (±6.6) years; 83.6% were under 5 years of age, and 45.8% were detected from male patients. The RV prevalence declined by approximately two-thirds, from 16.3% in the pre-vaccine era (2008–11 seasons) to 5.4% in the post-vaccine era (2015–20 seasons) as shown in Figure 1 and Table 1. As presented in Table 1, RV prevalence decreased significantly in children (1–9 years of age), yet adults and otherchildren groups (<1 year and 10–16 years of age) showed a different aspect. Analyzing monthly distribution by the number of typed samples, the RV seasons usually began in January and ended in May over the study period, and the peak occurred in February during the pre-vaccine period, but the peak was in March during the transitional and post-vaccine periods.

### 3.1. Changes in Distribution of the RV Genotypes Following Vaccine Introduction

**Figure 2:**
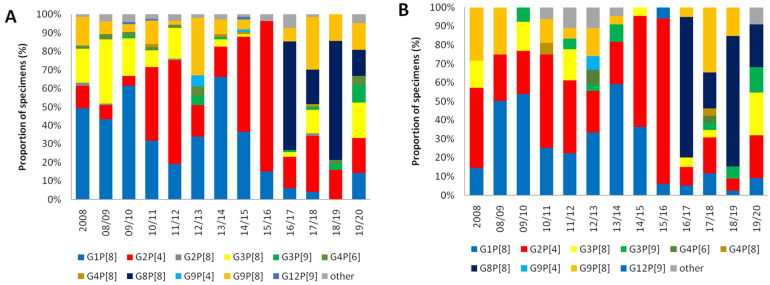
Temporal distribution of rotavirus genotypes identified by age <5 years of age (**A**) and ≥5 years of age (**B**) over the course of the study in Korea, 2008–2020. The proportion of each rotavirus genotypes identified is shown as a 100% stacked bar chart.

**Figure 3:**
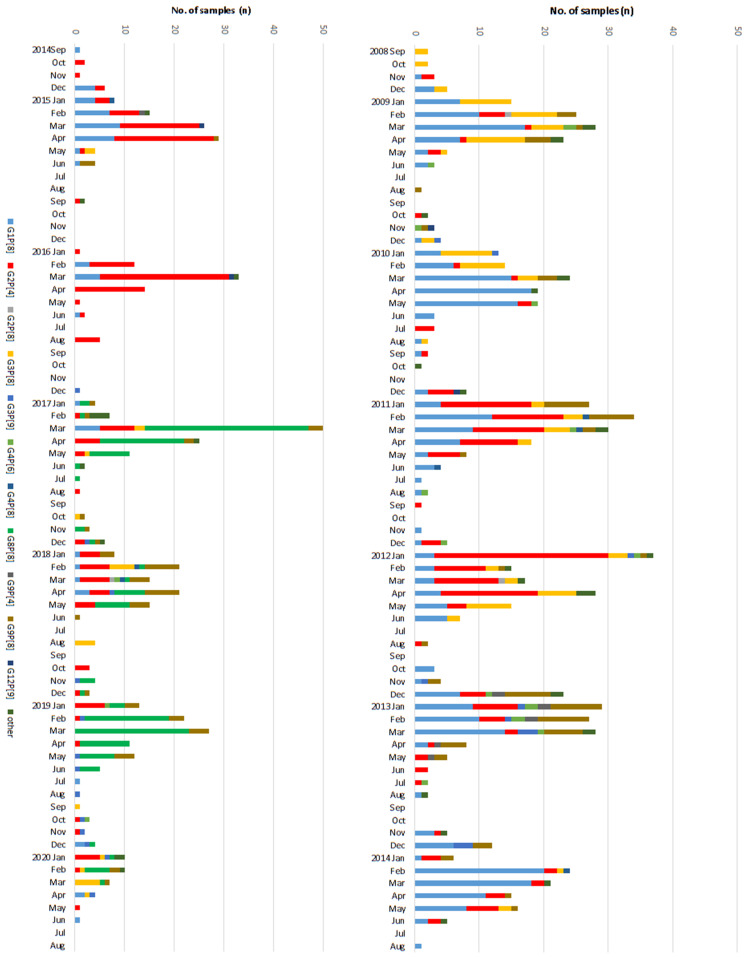
Temporal distribution of rotavirus genotypes identified per month over the course of the study in Korea, 2008–2020. The proportion of each rotavirus genotypes identified is shown as a stacked bar chart.

Of the 2919RV positive samples, 1386 samples (467 samples in pre-vaccine, 564 samples in transitional, 355 samples in post-vaccine period) were further analyzed for VP4 and VP7. Among the total 467 RV cases in the pre-vaccine period, G1P[8] was the most common genotype with 188 (40.3%) cases followed by G2P[4] (18.0%), G3P[8] (17.3%), and G9P[8] (9.0%), etc. In the transitional period, the proportion of G2P[4] (38.7%) became the dominant genotype and G1P[8] was still commonly identified in 35.0% of RV cases. In contrast, the novel genotype G8P[8] (157 cases, 44.2%) was predominant in the post-vaccine period, with reduced detection of G1P[8] (5.1%), G2P[4] (17.7%), and G3P[8] (6.2%). G9P[8] (15.2%) was also frequently detected in the post-vaccine period compared to the pre-vaccine period. Other genotypes identified were G2P[8] (0.3%), G3P[9] (1.8%), G4P[6] (1.4%), G4P[8] (0.5%), G9P[4] (0.6%), and G12P[9] (0.3%) over the study period. Rarer genotypes <3 RV cases, grouped as ‘other’, were G1P[4], G3P[4], G3P[19], G4P[2], G8P[6], G9P[6], G11P[25], and G12P[6] over the study period. In sum, during 2008–15 seasons (pre-vaccine and transitional period), G1P[8] and G2P[4] showed fluctuation patterns such as being competitive antagonists to each other as depicted in Figure 2. Then, suddenly, the novel G8P[8] became the major genotype in the post-vaccine period. Yet, no single genotype was dominant in the last 2019–20 season (Figure 3).

Throughout the study period, overall distribution of RV genotype showed little age-specific variations (Figure 2). G4P[6] was predominant in children <12 months of age in the pre-vaccine period, but the total number of positive RV cases of this age group was too small to give a single meaning. G1P[8] and G8P[8] were the most common genotypes for each age group in pre-vaccine and post-vaccine ages, respectively.

### 3.2. Differences of the RV Genotypes by Vaccine Status

**Figure 4:**
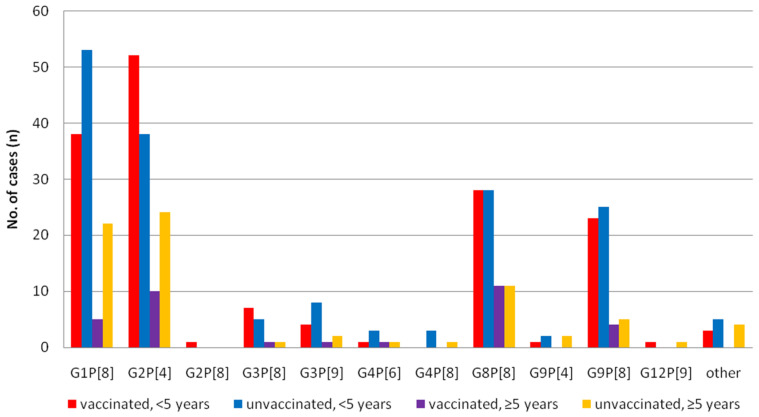
Distribution of rotavirus genotypes by age group and vaccination status in Korea.

**Figure 5:**
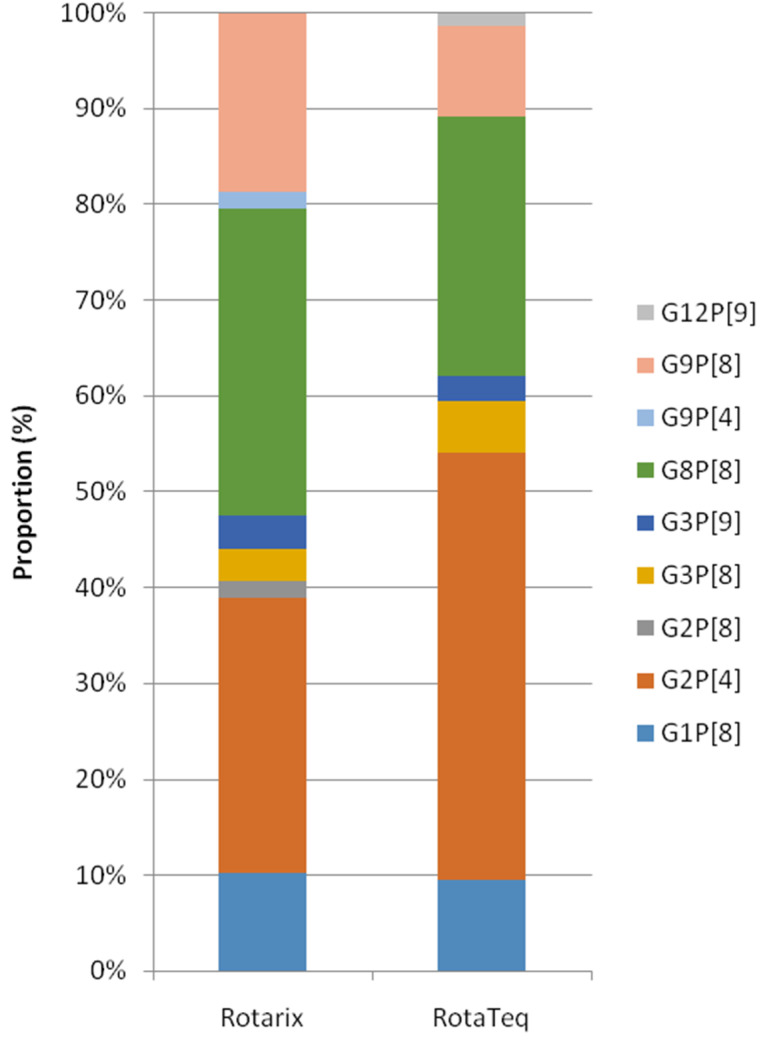
Distribution of rotavirus genotypes by the type of vaccine; Rotarix and RotaTeq in Korea, during post-vaccine period.

Since 2013, 7592 patient records clearly stated the vaccination status. Of these, 248 (5.9%) samples out of 4211 vaccinated patients and 362 (10.7%) out of 3381 unvaccinated patients were ELISA positive (*p* = 0.00, χ^2^ test). Among 610 RV positive samples, 436 cases (<5 years old; 329, older group; 107) were further analyzed (Figure 4). Reduction of G1P[8] after vaccination was evident in all age groups (*p* = 0.05, χ^2^ test), and G2P[4] became dominant in children (<5 years) group (*p* = 0.04, χ^2^ test). Other major genotypes such as G8P[8] and G9P[8] were equally distributed regardless of age and vaccination status.We also analyzed the diversity of genotypes according to the types of vaccines used from a total of 133patients (Rotarix; 59, RotaTeq; 74) in the post-vaccine period, whose vaccine types were recorded. G2P[4] (28.8%) and G8P[8] (32.2%) were two major genotypes in the Rotarix group and G2P[4] (44.6%) and G8P[8] (27.0%) were also identified in the RotaTeq group. No statistical differences were observed between the two vaccine groups, though there were numerical differences such as G9P[8] in the Rotarix group, at 18.6%, and in the RotaTeq group, at 9.5% (Figure 5).

## 4. Discussion

The efficacy of RV vaccines has been well studied since its licensure, and ranges between 54% and 89%, depending on the age group, different child mortality levels, and vaccine status such as vaccine type, number of doses, etc. [8]. In addition, the RV vaccine has had a substantial effect on morbidity, mortality, hospitalization, and social costs, etc. caused by rotaviral diarrhea and its complications [9,11]. For instance, in Austria, the annual number of hospitalizations by RV reduced from 74 to 88% in children < 18 years [13]. A national hospital database study in Spain revealed a 43% reduction in RV hospitalization [14]. Burnett et al. reported that the mortality in children <5 years was reduced 42% in countries with medium and high mortality [8]. After the vaccine introduction in the US, 53% reductions in acute gastroenteritis (AGE)-related hospitalizations were observed, and over $1.2 billion was saved from 2008 to 2013 [15]. During 13 RV seasons, we observed a gradual decrease in RV positive rates from 14.5% in the pre-vaccine period to 8.2% and 5.5% in the transient and post-vaccine periods, respectively. Only 2.2% of AGE patients contributed to RV in our last study season, 2019/20.

Our results also showed a delayed RV peak from February to March with a blunt shape. Yet, this postponement does not seem to be just because of the vaccine effect, as Suzuki et al. reported that peak rotavirus activity had shifted from January to March during the 21 seasons to 2003 in Japan for unknown reasons [16]. On the other hand, after the introduction of the RV vaccine, delayed onset, peak of RV season, and blunting of seasonal peaks were observed compared to the pre-vaccine period in US [17,18].

The distribution of rotavirus genotypes may differ by geographical area all across the world, time period, climates, and socio-economic status, etc. [19]. Long-term surveillance during the initial rotavirus vaccine period in European countries such as France and the UK revealed G1P[8] was a major circulating strain during 2007–14 [20], whereas G3P[8] was predominant in the United States, China, and Japan [3,21,22]. Meanwhile, before the introduction of RV vaccine, G1P[8] was the most prevalent strain, followed by G3P[8] and G4P[6] in Seoul, Korea [23]. A few studies reported that G9P[8] was prevalent in the initial years (2007–09) of RV vaccine, then G1P[8] resurged during 2008–10 and again in 2011–13. G3P[8] and G2P[4] were predominant during 2010–11 and 2013–15 [24,25,26]. Genetic analysis revealed that multiple interspecies re-assortment events might have contributed to the emergence of G2P[4] during 2013–15 in South Korea [26]. A gradual decline of G1P[8] and G3P[8] and an increase of the infrequent G2P[4] in the pre-vaccine period were evident in this study.

During the transient period, G1P[8] resurged from the 2011/12 season to the 2013/14 season as G2P[4] diminished, and then handed over the top position to G2P[4] in the 2015/16 season. It was not until the 2016–17 season that something other than G1P[8] or G2P[4] became the dominant genotype, though G2P[4] still held a seat in the 2019/20 season. Predominance of the G2P[4] strain in the post-vaccine era was depicted in many countries, including Austria, Belgium, the UK, Finland, Australia, Brazil and Venezuela, where Rotarix was implement as NIPs [3,22,27]. However, in countries using RotaTeq or the mixed vaccine including the United states, Korea, and Japan, different or no consistent dominance pattern with elevated G2P[4] detection was observed [3]. For instance, common genotypes of the pre-vaccination era like G1P[8] and G3P[8] was reduced after the introduction of RV vaccine, whereas unusual G2P[4], G9P[8], and G8P[8] strains emerged and reemerged in the post-vaccination period in Japan [28,29]. In the US, G3P[8] and G12P[8] dominated in 2009–11 and 2012–13, respectively [21]. Actually, similar increases of G2P[4] strains have been reported even in countries without the vaccination [4], and annual fluctuations in G2P[4] prevalence seemed to occur naturally in disregard of the type of vaccination programs; Rotarix, RotaTeq, or Mixed type [30]. Thus, whether the diversity and distribution of RV genotypes represent vaccine pressure or natural seasonal fluctuations seem to be still unclear.

Interestingly, an unusual G8P[8] emerged as the dominant strains in the 2016/17 season, which was detected for the first time in Gwangju city, Korea. G8P[8] strains have occasionally been described in the post-vaccine era in Japan, Singapore, Thailand, Vietnam, and Argentina [31,32,33,34]. G8P[8] was first reported in 2004–06 in Korea and then detected in a Rotarix-vaccinated child in 2018 [24,35]. Yet, the surge of G8P[8] to a predominant level in the post-vaccine period was a new finding in this study. Surveillance data in Vietnam during 2012–2015 depicted a considerable surge in G8P[8] prevalence, which increased from 1% in 2014 to 31% in 2015 [36]. A rare G8P[8] strain was detected at a substantial level for the first time in the Czech Republic during 2016–2019, which was the first time that G8P[8] strains were detected at a high rate in Central Europe [37]. Phylogenetic analysis of the G8 VP7 segment revealed that the Czech bovine–human DS-1-Like G8P[8] strains clustered with strains described in Vietnam, Singapore, Thailand, and Japan. Lee, SK et al., reported that G8P[6] strains from Korean neonates were expected to be derived from reassortment events between the G8 of G8P[8] strains in the Asian region [38]. The emergence and surge of G8P[8] in Gwangju, Korea may be attributed to importation of these G8P[8] strains. Additional analysis of G8 VP7 gene segments of the rotaviruses will improve our understanding.

There are several shortcomings in this study. First, our data may not be representative of the whole country, as it only included sentinel surveillance data from the south-east region of Korea. Second, we also lacked the vaccination status in most cases because the enrollment of vaccine status depends on one’s voluntariness, which may help assessthe effect of each RV vaccines. Third, patients with mild symptoms, particularly in older children and adults, who might possibly get natural immunity from previous rotavirus infections, do not need medical help. Therefore, our data are mostly of young children under 5 years of age with moderate to severe symptoms. However, we have had enough samples spanning 13 seasons to analyze the prevalence of RV cases and diversity, seasonal pattern, and fluctuations of RV genotype.

## 5. Conclusions

We have evaluated the long-term impact of RV vaccination on prevalence, seasonality, and genotype distribution in Korea for 13 seasons, 2008–20. The substantial decline of G1P[8] prevalence after RV vaccine introduction, especially by Rotarix, was also observed in this study. The emergence and reemergence of G1P[8], G3P[8], and G2P[4] RV strains were evident over the whole study period. The emergence and surge of G8P[8] in the post-vaccine period, which is speculated to be closely related to the strains reported in some countries such as the Czech Republic, Vietnam, Singapore, Thailand, and Japan, were an interesting point to note. All things considered, it is very likely that not only natural/seasonal shifts, but also vaccine-related selection pressure has resulted in the diversity of rotavirus genotype since RV vaccination, and caution is required when interpreting temporal changes in RV genotype dynamics. The continuous surveillance on the genotypes of RVA will be needed to monitor circulating wild-type strains, as well as rotavirus genotype constellations and whole-genome characteristics, to understand rotavirus epidemiology and their evolutionary patterns.

## Figures and Tables

**Table 1:** Rate of rotavirus detection by age group and prevalence of distinct RVA genotypes in the pre-, transitional, and post-vaccination periods in Korea, 2008–2020. *p*-values were calculated for the pre-vaccine period and the post-vaccine period.

	Pre-Vaccine	Transitional	Post-Vaccine	*p*-Value
Age group				
<1 years	35/1145 (3.1%)	20/955 (2.1%)	28/1291 (2.2%)	0.17
1 years	507/3567 (14.2%)	130/2301 (5.6%)	75/1539 (4.9%)	<0.01
2 years	619/2138 (29%)	186/1174 (15.8%)	78/927 (8.4%)	<0.01
3 years	300/1024 (29.3%)	111/686 (16.2%)	65/697 (9.3%)	<0.01
4 years	140/667 (21%)	90/513 (17.5%)	58/575 (10.1%)	<0.01
5–9 years	157/1518 (10.3%)	95/1103 (8.6%)	115/1855 (6.2%)	<0.01
10–16 years	19/657 (2.9%)	18/408 (4.4%)	11/734 (1.5%)	0.07
adults (>17 years)	4/139 (2.8%)	6/139 (3.2%)	17/500 (3%)	0.76
Total	1792/11009 (16.3%)	676/7567 (8.9%)	451/8326 (5.4%)	<0.01
Roravirus genotypes				
G1P[8]	188/467 (40.3%)	186/564 (33.0%)	18/355 (5.1%)	<0.01
G2P[4]	84/467 (18.0%)	218/564 (38.7%)	63/355 (17.7%)	0.93
G3P[8]	81/467 (17.3%)	27/564 (4.8%)	22/355 (6.2%)	<0.01
G8P[8]	-	-	157/355 (44.2%)	<0.01
G9P[8]	42/467 (9.0%)	51/564 (9.0%)	54/355 (15.2%)	<0.01

## Data Availability

All datasets generated for this study are included in the article.
